# Depletion of follicular B cell-derived antibody secreting cells does not attenuate angiotensin II-induced hypertension or vascular compliance

**DOI:** 10.3389/fcvm.2024.1419958

**Published:** 2024-05-31

**Authors:** Hericka Bruna Figueiredo Galvao, Maggie Lieu, Seyuri Moodley, Henry Diep, Maria Jelinic, Alexander Bobik, Christopher G. Sobey, Grant R. Drummond, Antony Vinh

**Affiliations:** ^1^ Centre for Cardiovascular Biology and Disease Research (CCBDR), La Trobe Institute of Medical Science (LIMS), La Trobe University, Melbourne, VIC, Australia; ^2^ Department of Microbiology, Anatomy, Physiology & Pharmacology, School of Agriculture, Biomedicine and Environment, La Trobe University, Melbourne, VIC, Australia; ^3^ Victorian Heart Institute, Monash University, Clayton, VIC, Australia; ^4^ Baker Heart and Diabetes Institute, Prahran, VIC, Australia; ^5^ Department of Immunology, Monash University, Clayton, VIC, Australia; ^6^ Center for Inflammatory Diseases, School of Clinical Sciences, Faculty of Medicine, Nursing and Health Sciences, Monash University, Clayton, VIC, Australia

**Keywords:** hypertension, B cells, plasma cells, antibodies, angiotensin II, Blimp-1

## Abstract

**Introduction:**

Marginal zone and follicular B cells are known to contribute to the development of angiotensin II-induced hypertension in mice, but the effector function(s) mediating this effect (e.g., antigen presentation, antibody secretion and/or cytokine production) are unknown. B cell differentiation into antibody secreting cells (ASCs) requires the transcription factor Blimp-1. Here, we studied mice with a Blimp-1 deficiency in follicular B cells to evaluate whether antibody secretion underlies the pro-hypertensive action of B cells.

**Methods:**

10- to 14-week-old male follicular B cell Blimp-1 knockout (FoB-Blimp-1-KO) and floxed control mice were subcutaneously infused with angiotensin II (0.7 mg/kg/d) or vehicle (0.1% acetic acid in saline) for 28 days. BP was measured by tail-cuff plethysmography or radiotelemetry. Pulse wave velocity was measured by ultrasound. Aortic collagen was quantified by Masson's trichrome staining. Cell types and serum antibodies were quantified by flow cytometry and a bead-based multiplex assay, respectively.

**Results:**

In control mice, angiotensin II modestly increased serum IgG3 levels and markedly increased BP, cardiac hypertrophy, aortic stiffening and fibrosis. FoB-Blimp-1-KO mice exhibited impaired IgG1, IgG2a and IgG3 production despite having comparable numbers of B cells and ASCs to control mice. Nevertheless, FoB-Blimp-1-KO mice still developed hypertension, cardiac hypertrophy, aortic stiffening and fibrosis following angiotensin II infusion.

**Conclusions:**

Inhibition of follicular B cell differentiation into ASCs did not protect against angiotensin II-induced hypertension or vascular compliance. Follicular B cell functions independent of their differentiation into ASCs and ability to produce high-affinity antibodies, or other B cell subtypes, are likely to be involved in angiotensin II-induced hypertension.

## 1 Introduction

Hypertension is a major risk factor for cardiovascular disease and has been identified by the World Health Organisation as the leading risk factor for the global burden of disease, accounting for 10.8 million deaths annually (1). Hypertension is a multifactorial condition due to dysregulation of one or more of the body's blood pressure-regulating systems. These include: the heart, where increased sympathetic activity leads to an increase in cardiac output; the vascular system where vasoconstriction and reduced arterial compliance lead to increased pulse pressure and peripheral vascular resistance; and altered salt/fluid handling by the kidneys can increase plasma volume (2). While therapies targeting these “classical” mechanisms of hypertension are effective at reducing blood pressure, they fail to lower blood pressure to target levels for patients with uncontrolled (∼50% of hypertensive patients) (3) or resistant hypertension (∼10% of hypertensive patients on 3 or more therapies concurrently) (4), thus suggesting the involvement of other pathological mechanisms.

There is strong evidence for an involvement of the immune system in hypertension (2, 5). In the early 1970s, it was established that hypertensive patients have elevated serum antibody titres (6–9) and inflammatory biomarkers (10–12). Moreover, IgG antibodies are known to accumulate in the vasculature and kidneys during hypertension (13–15) and IgG autoantibodies against α_1_-adrenoceptors and β_1_-adrenoceptors have also been identified in hypertensive patients (16–20). Given that antibodies are produced exclusively by B cells, these clinical observations point to a potential role for B cells in the development of hypertension. In support of this, preclinical studies have shown that angiotensin II-induced hypertension in mice is associated with B cell activation, increased serum IgG titres (21), as well as the formation of germinal centre B cells (22) and memory B cells (23). The depletion of marginal zone and follicular B cells, either genetically or pharmacologically with an anti-CD20 monoclonal antibody, blunts the pressor response to angiotensin II (21). Furthermore, the pharmacological neutralisation or genetic depletion of interleukin-21, produced by T follicular helper cells to stimulate the formation of germinal centre reactions, reduced blood pressure, endothelial dysfunction and vascular inflammation in angiotensin II-infused mice (22).

Despite such direct evidence for a pro-hypertensive role for B cells, it remains unknown whether increased IgG antibody production during hypertension is required for these effects or is merely a marker of increased B cell activity. Indeed, antibody production is only one of several mechanisms by which B cells can influence immunity and inflammation. Other mechanisms including antigen presentation and cytokine production (24) could therefore also contribute to pro-hypertensive actions of B cells. In other diseases, the presentation of antigens by B cells has been shown to promote systemic lupus erythematosus (SLE) (25) and atherosclerosis (26). Similarly, B cell secretion of tumour necrosis factor-alpha (TNF-α) (27) promoted atherosclerosis, while interleukin-6 and interferon-gamma (IFN-γ) secretion induced T cell proliferation, Th17 polarisation and reductions in regulatory T cells in autoimmune encephalomyelitis (28) and proteoglycan-induced arthritis (29).

High-affinity IgG antibodies are mainly produced by follicular B cells following their activation within secondary lymphoid organs and commitment to an antibody secreting cell (ASC) fate (30). The terminal differentiation of B cells into ASCs requires the transcriptional repression of B cell-specific transcription factors (Pax5, Bach2 and Bcl6) (31) and the de-repression of the B lymphocyte-induced maturation protein-1 (Blimp-1) transcription factor, encoded by the *Prdm1* gene (32, 33). While Pax5 drives the expression of genes associated with antigen processing and presentation, Blimp-1 drives the expression of genes required for the high rate of antibody secretion that is characteristic of ASCs (31). In the present study, we used an established Cre-Lox animal model to selectively inhibit *Prdm1* gene activity in follicular B cells (26) (hereafter referred to as FoB-Blimp-1-KO mice). This model was previously shown to prevent follicular B cell differentiation into ASCs and reduce total plasma IgG (26). Thus, this approach allows us to investigate the effect of follicular B cell-derived antibody depletion, without disrupting other B cell functions, on the development of hypertension, vascular and cardiac complications in response to angiotensin II. Here, we showed a reduction in serum IgG1, IgG2a and IgG3 levels that is consistent with the functional impairment of follicular B cell differentiation into ASCs. Nevertheless, FoB-Blimp-1-KO mice showed comparable hypertension, cardiac hypertrophy and vascular fibrosis to control mice when both were infused with angiotensin II. Hence, our findings suggest that the previously reported pro-hypertensive actions of B cells occur independently of the differentiation of follicular B cells into ASCs and the subsequent production of IgG antibodies. Hence, it would seem that other B cells processes such as antigen presentation (25, 26) and/or cytokine production (27–29, 34–38), or alternatively antibody production from other B cell subsets, may instead contribute to the development of hypertension in response to angiotensin II infusion.

## 2 Materials and methods

### 2.1 Animals and ethics

The animals and procedures were approved by the La Trobe University Animal Ethics Committee (AEC 16-93), the Monash University Animal Research Platform Ethics Committee (MARP/2016/077) and Institutional Biosafety Committee (GM16-25). All animal experiments were performed in accordance with the Australian Code for the Care and Use of Animals for Scientific Purposes 8th Edition 2013 (updated 2021).

To generate mice with a deficiency in follicular-derived ASCs, Cre recombinase under the control of the CD23 (*Fcer2a*) promoter that is expressed in follicular B cells (39) was used to truncate the Blimp-1 (*Prdm1*) transcription factor (26). Control (*Prdm1*^fl/fl^) and FoB-Blimp-1-KO (*Fcer2a-Cre*^+/−^*Prdm1*^fl/fl^) mice on a C57BL6 background were bred at the La Trobe Animal Research and Teaching Facility (Bundoora, Australia) and the Animal Research laboratories (Clayton, Australia). Mice were housed in temperature (22 ± 2 °C) and humidity (55 ± 15%) controlled rooms with 12-hour light/dark cycles in individually ventilated cages with access to food and water *ad libitum*. To build upon our previous study (21) showing the global pharmacological and genetic depletion of B cells in male mice blunted the pressor response of angiotensin II, the present follow up study used 10- to 14-week-old male controls and FoB-Blimp-1-KO mice, with initial weights of 27.5 ± 0.4 g (mean ± S.E.M).

### 2.2 Genotyping

B cells were isolated from spleens of untreated FoB-Blimp-1-KO and control mice using a B cell isolation kit (Miltenyi Biotec, Australia). The Wizard® SV Genomic DNA purification system (Promega, USA) was used to purify genomic DNA from B cell samples. Genomic DNA was amplified by PCR using primers specific for Blimp-1 or Cre recombinase using a thermal cycler (My CyclerTM, Biorad, USA). PCR products and the DNA standard, HyperladderTM 100 bp (Bioline, Australia) were added into wells of a 2% agarose gel in Tris/Borate/EDTA (TBE) buffer solution with 0.01% SYBR® Safe (Thermo Fisher Scientific, USA). Images were acquired using a ChemiDocTM MP Imaging system (Biorad, USA), as shown in Supplementary Figure S1. The expected sizes of the bands for Blimp-1 for each mouse genotype were: 765 bp for the floxed allele and 646 bp for exon 6-deleted (truncated) allele (40). The expected bands for Cre recombinase-expressing mice were: 750 bp for heterozygous Cre-positive/wild-type and N/A for wild-type (non Cre-expressing).

### 2.3 Blinding, randomisation and sample sizes

The primary investigator was blinded to the genotype and treatment of the animals for the duration of the experimental protocol and data analysis. Treatment allocations were randomised using an electronic coin flip. Power calculations indicated that a sample of size of *n *= 9 was necessary to identify a 25% mean effect change in systolic blood pressure (SBP) with 80% power and 10% standard deviation (*P* < 0.05). All experimental groups had sample sizes of 9–10 animals.

### 2.4 Induction of hypertension

Mice, randomly allocated to the hypertensive group, were infused with angiotensin II (0.7 mg/kg/d, *s.c.*) for 28 days, while those allocated to the normotensive group were infused with a vehicle solution containing 0.1% acetic acid in saline. This was achieved by surgically implanting pre-filled osmotic minipumps (Alzet Model 2004, USA) dorsally between the scapulae under isoflurane anaesthesia (2%–3% at 0.2–0.5 L/min (41). Mice received three doses of an analgesic (5 mg/kg carprofen, *s.c.*) at 0, 24 and 48 h post-surgery.

### 2.5 Blood pressure measurement

BP measurements were acquired via tail-cuff plethysmography using the MC4000 Multichannel system (Hatteras Instruments, USA) or via radiotelemetry using a telemeter probe (Model TA11PA-C10, Data Sciences International, USA). For tail-cuff measurements, mice were allowed to acclimatise to the procedure for 1 week prior to the acquisition of baseline and post-surgery systolic BP measurements (42). For telemetric monitoring, mice were allowed at least 10 days of recovery following probe implantation (43) prior to measurement of baseline BP and induction of hypertension.

### 2.6 Tissue harvesting

At day 28, mice were euthanised by CO_2_ asphyxiation followed by diaphragmatic puncture. Blood samples were collected by left ventricular cardiac puncture and placed immediately on ice until further processing. Mice were then perfused with PBS and their spleen and bone marrow were harvested and transferred to 1.5 ml tubes filled with ice cold PBS for subsequent flow cytometric analysis.

### 2.7 Flow cytometry

Spleen and bone marrow samples were prepared into single cell suspensions (13, 41, 42). Briefly, tissue samples were mechanically digested and incubated with a red blood cell lysis buffer (0.15 M NH_4_Cl, 0.01 M KHCO_3_, 6.0 mM EDTA, dH_2_O) for 5 min. The samples were then pelleted by centrifugation and resuspended in PBS at 1:1 for automated cell counting using trypan blue and a Countess™ Automated Cell Counter (Invitrogen). The samples were then diluted to obtain a final live cell concentration of 10^7^ single cells per ml of FACS buffer [0.5% bovine serum albumin (BSA; Sigma-Aldrich, USA) in PBS].

Approximately 2 × 10^6^ live cells per sample were loaded into 96-well microplates and incubated with a cocktail of fluorescently labelled antibodies against various immune and B cell surface markers (Table 1). The cells were then permeabilised and further stained with an intracellular anti-Blimp-1 fluorescently-labelled antibody, prior to being fixed in 1% formalin in FACS buffer (42). Stained samples were then run on a BC CytoFLEX S flow cytometer (Beckman Coulter, USA) where up to ∼10^6^ live leukocytes were counted. Data were analysed using FlowJo Software (version 10.8.1 Tree Star Inc., USA).

**Table 1:** Surface and intracellular target markers used for flow cytometry.

Target	Clone	Fluorophore	Dilution
anti-CD38	90	PE-Cy7	1:500
anti-CD23	B3B4	FITC	1:500
anti-CXCR4	2B11	PE-610	1:500
anti-Sca-1	D7	BV605	1:500
anti-CD138	281-2	BV650	1:500
anti-CD98	RL388	A647	1:500
anti-CD19	6D5	A700	1:500
anti-B220	RA3-6B2	BV421	1:500
anti-Blimp-1 (intracellular)	5E7	PE	1:1,000

### 2.8 Gating strategy

Spleen and bone marrow ASCs were quantified using a modified gating strategy adopted from Wilmore et al. (44), where ASCs were identified as CD138^hi^Sca-1^hi^Blimp-1^+^ cells and then further subdivided into plasmablasts (CD138^hi^Sca-1^hi^Blimp-1^+^B220^+^) and plasma cells (CD138^hi^Sca-1^hi^Blimp-1^+^B220^−^) (42) (Supplementary Figure S2). Innate-like B-1 (CD19^+^B220^−^), CD23^+^ and CD23^−^ conventional B-2 (CD19^+^B220^+^) B cells were additionally quantified to identify potential changes to the B cell compartment in Blimp-1 knockout mice vs. controls as shown in Supplementary Figure S2.

### 2.9 Quantification of serum antibody titres

Blood samples were allowed to clot at room temperature and then centrifuged at 2,000×*g* for 10 min at 4 °C. The supernatant was collected and stored in 1.5 ml tubes at −80 °C for further analysis. Frozen serum samples were thawed and their immunoglobulin isotypes were analysed using a fluorescent bead-based assay panel (LEGENDplex™ Mouse Immunoglobulin Isotyping Panel; BioLegend, USA). Briefly, serum samples were diluted (1:50,000), combined with the bead and assay buffer mixture, and incubated in V-bottom 96-well microplates for 2 h. Biotinylated detection antibodies were then added to all microplate wells and allowed to incubate for 1 h before the addition of streptavidin-phycoerythrin (SA-PE). The samples were read on a flow cytometer (BC CytoFLEX S flow cytometer; Beckman Coulter, USA) and serum IgG1, IgG2a, IgG2b, IgG3, IgA and IgM concentrations were inferred from corresponding 7-point standard curves using the LEGENDplex™ Data Analysis Software Suite.

### 2.10 Histochemical staining

Freshly isolated aortas were immersed in Tissue-Tek® O.C.T (Sakura Finetek, Japan), snap frozen and cut into 10 *μ*m sections using a cryostat (CM1850, Leica Microsystems, Germany). Sections were stained with Masson's trichrome and imaged by an Aperio Scanscope AT Turbo scanner (Leica Biosystems, Australia). Analyses were performed blinded to the treatment group using the Aperio Imagescope software (Leica Biosystems, Australia). Aortic collagen deposition was quantified as the average total adventitial area positively stained for Masson's trichrome in two non-serial sections per animal.

### 2.11 Measurement of vessel stiffness

Pulse wave velocity (PWV, a measure of aortic stiffness) was measured using high-resolution ultrasound imaging (Vevo 2100, FUJIFILM Visualsonics Inc., Canada) (21). Briefly, mice were anaesthetised with 1.5% isoflurane and placed on a heated platform. Fur was removed from the abdomen with Nair® and the exposed skin was coated with ultrasound transmission gel (Aquasonic, USA). Pulsed-wave doppler images and EKV™ retrospective acquired B-Mode images were obtained from longitudinal sections of abdominal aortas (suprarenal) using a MS-400 ultrasound transducer. Data were analysed using the VevoLab and VevoVasc software (FUJIFILM Visualsonics Inc., Canada) and the ln(D)-V loop method was used to obtain measures of aortic PWV (45).

### 2.12 Statistical analyses

The D'Agostino & Pearson test was used to check data distribution and a ROUT test set to a False Discovery rate of 1% was used to identify outliers. All analyses were performed with GraphPad Prism 9.4.0 software (GraphPad Software, San Diego, California, USA) and hypothesis tests with an alpha probability of less than 5% (*P* < 0.05) were considered statistically significant. Tail-cuff BP datasets were analysed using a mixed effects model with Geisser-Greenhouse correction, followed by Tukey's multiple comparisons test, while flow cytometry datasets were analysed using a two-way ANOVA followed by Tukey's multiple comparisons test. Radiotelemetry data were analysed using a repeated measures two-way ANOVA with Bonferroni's multiple comparisons test. PWV and Masson's trichrome data were analysed using Student's unpaired t-tests. Data are presented as the mean ± S.E.M. (Standard Error of the Mean).

## 3 Results

### 3.1 FoB-Blimp-1-KO mice were protected from angiotensin II-induced increases in serum IgG3 without changes in ASC numbers

**Figure 1:**
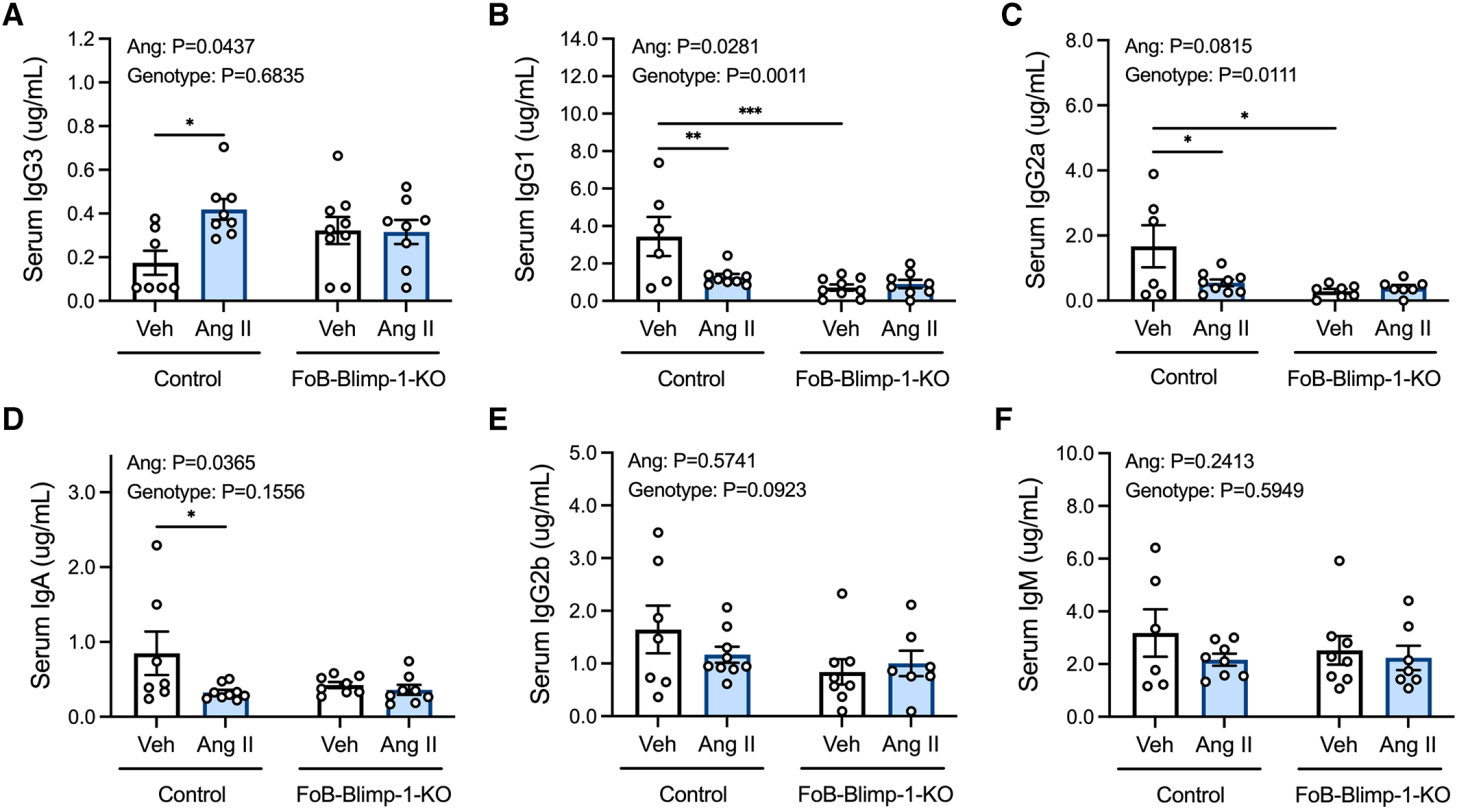
Effect of follicular B cell Blimp-1 truncation on serum antibody levels in response to angiotensin II. Serum samples were acquired from control and FoB-Blimp-1-KO mice for the simultaneous quantification of mouse IgG3 (**A**), IgG1 (**B**), IgG2a (**C**), IgA (**D**), IgG2b (**E**) and IgM (**F**) immunoglobulin isotypes using a multiplex bead-based assay. Data are represented as the mean ± standard error of the mean. *P value <0.05. **P value <0.01. ***P value <0.001. Treatment effects are indicated at the top of the graphs.

Serum IgG3 concentrations were approximately 2.4-fold higher in control mice infused with angiotensin II than in those infused with vehicle solutions (Figure 1A). By contrast, angiotensin II reduced IgG1, IgG2a and IgA in control mice (Figures 1B–D). FoB-Blimp-1-KO mice had lower basal levels of serum IgG1 and IgG2a but did not show a change in basal IgG3 levels or following angiotensin II infusion (Figures 1A–C). Serum IgG2b and IgM were unaffected by angiotensin II infusion and follicular Blimp-1 truncation (Figures 1E,F).

**Figure 2:**
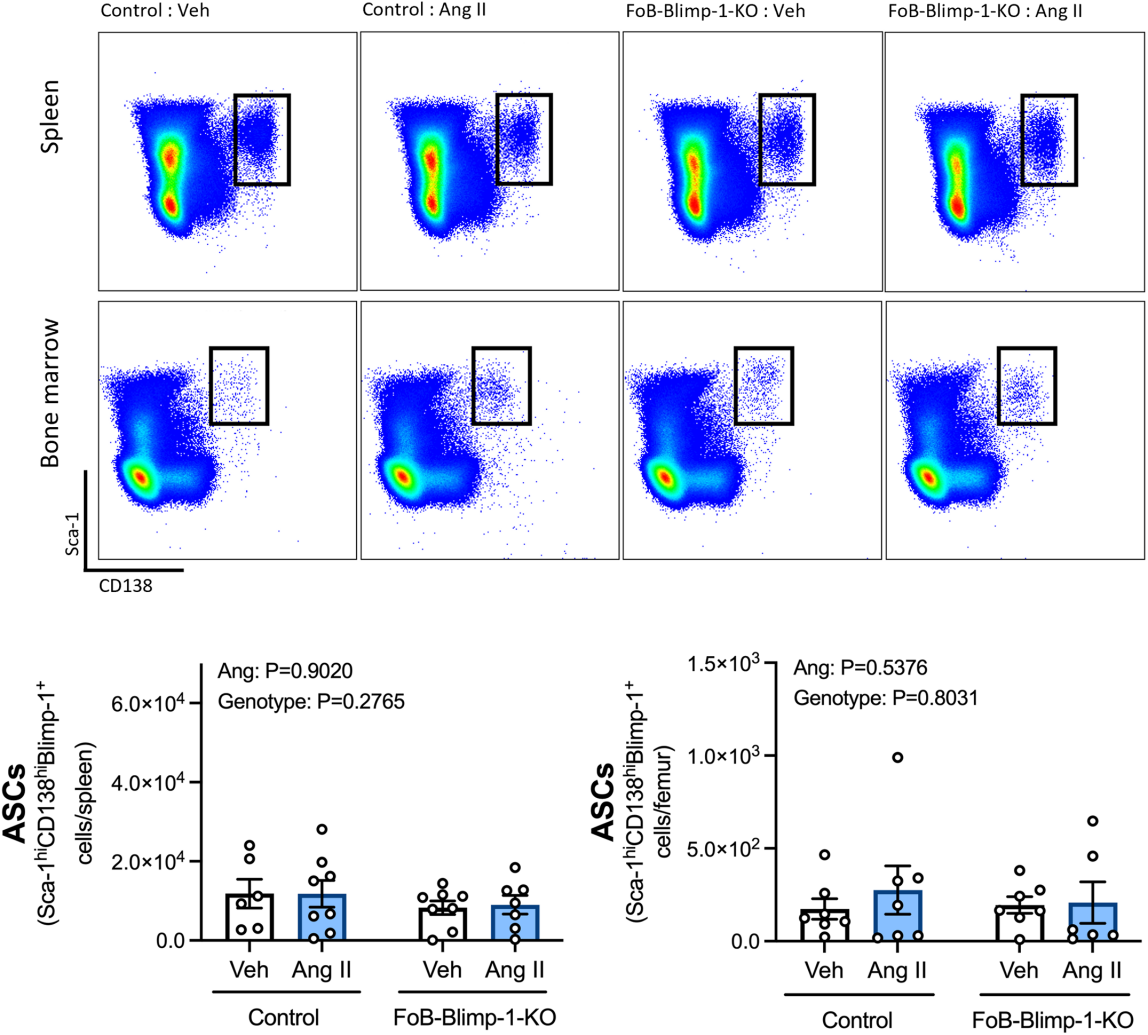
FoB-Blimp-1-KO mice have a similar number of B cells committed to an ASC fate as control mice in the spleen and bone marrow. Representative flow cytometry plots and bar graphs showing ASC numbers in control and FoB-Blimp-1-KO mice infused with either angiotensin II (0.7 mg/kg/day) or vehicle (0.5% NaCl, 0.1% acetic acid) solutions for 28 days. ASCs were identified as Sca-1^hi^CD138^hi^Blimp-1^+^ cells, gated from all live splenocytes or all live bone marrow-derived cells. Data are represented as the mean ± standard error of the mean of the total cell counts per spleen or per femur. Treatment effects are indicated at the top of the graphs.

**Figure 3:**
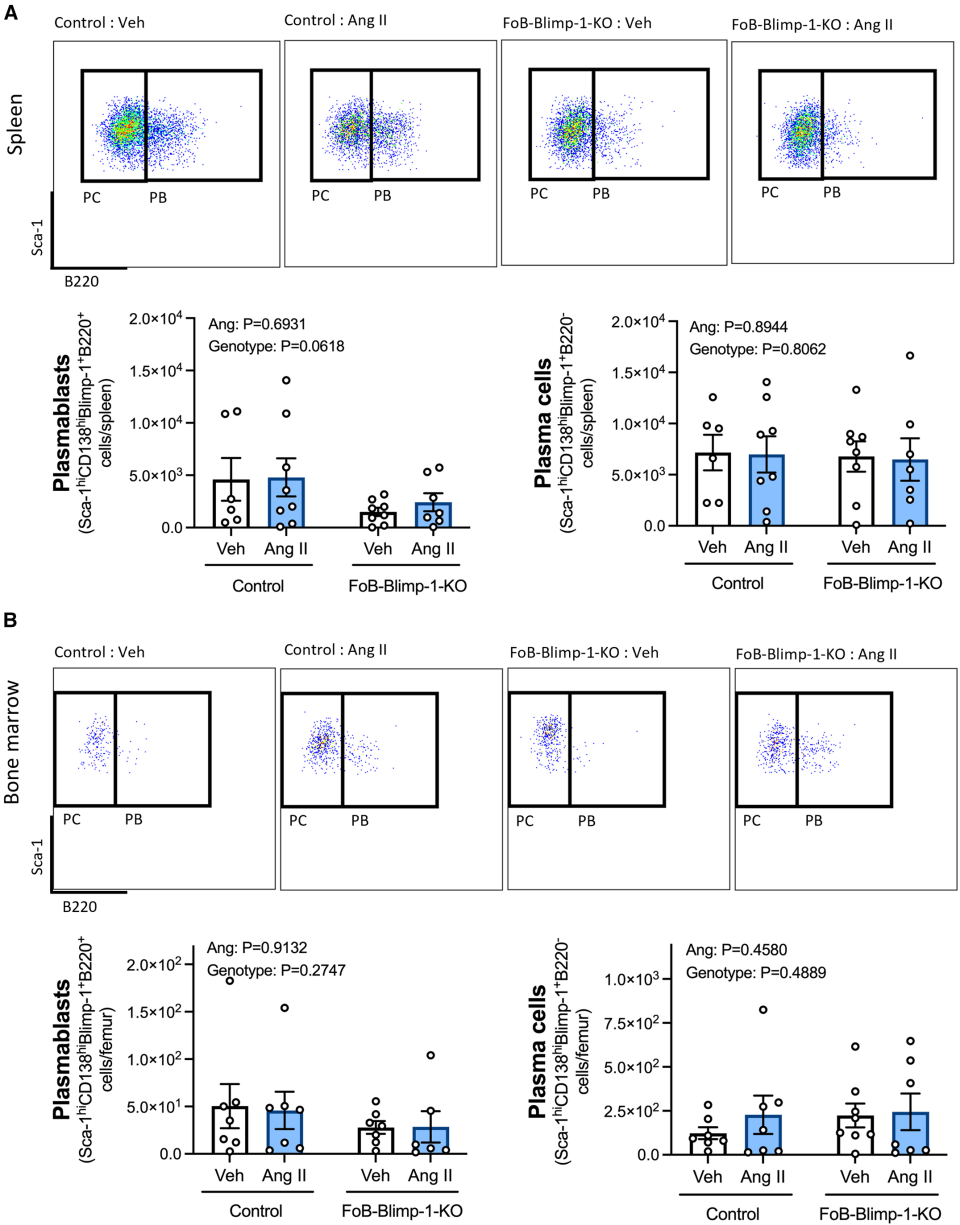
Neither a targeted Blimp-1 truncation nor angiotensin II infusion affected plasmablasts or plasma cells in the spleen or bone marrow. Representative flow cytometry plots and bar graphs showing plasmablast (PBs) and plasma cell (PCs) numbers in the spleen (**panel A**) and bone marrow (**panel B**) of control and FoB-Blimp-1-KO mice infused with either angiotensin II (0.7 mg/kg/day) or vehicle (0.5% NaCl, 0.1% acetic acid) solutions for 28 days. PBs were defined as B220+ and PCs as B220- cells gated on Sca-1hiCD138hiBlimp-1+ ASCs. Data are represented as the mean ± standard error of the mean of the total cell counts per spleen or per femur. Treatment effects are indicated at the top of the graphs.

Changes in serum antibody isotypes in FoB-Blimp-1-KO mice occurred independently of B cell commitment to an ASC fate in the spleen or bone marrow, as shown by the absence of change in total ASC numbers (Figure 2). Angiotensin II infusion also did not affect the total number of B cells committed to an ASC fate in either organ (Figure 2), nor the number of plasmablasts and plasma cells (Figure 3). As expected, neither Blimp-1 truncation nor angiotensin II infusion affected the B cell compartment in the spleen or bone marrow of control or FoB-Blimp-1-KO mice (Supplementary Figures S3 and S4).

### 3.2 FoB-Blimp-1-KO and control mice developed similar hypertension, cardiac hypertrophy, fibrosis and aortic stiffening in response to angiotensin II infusion

**Figure 4:**
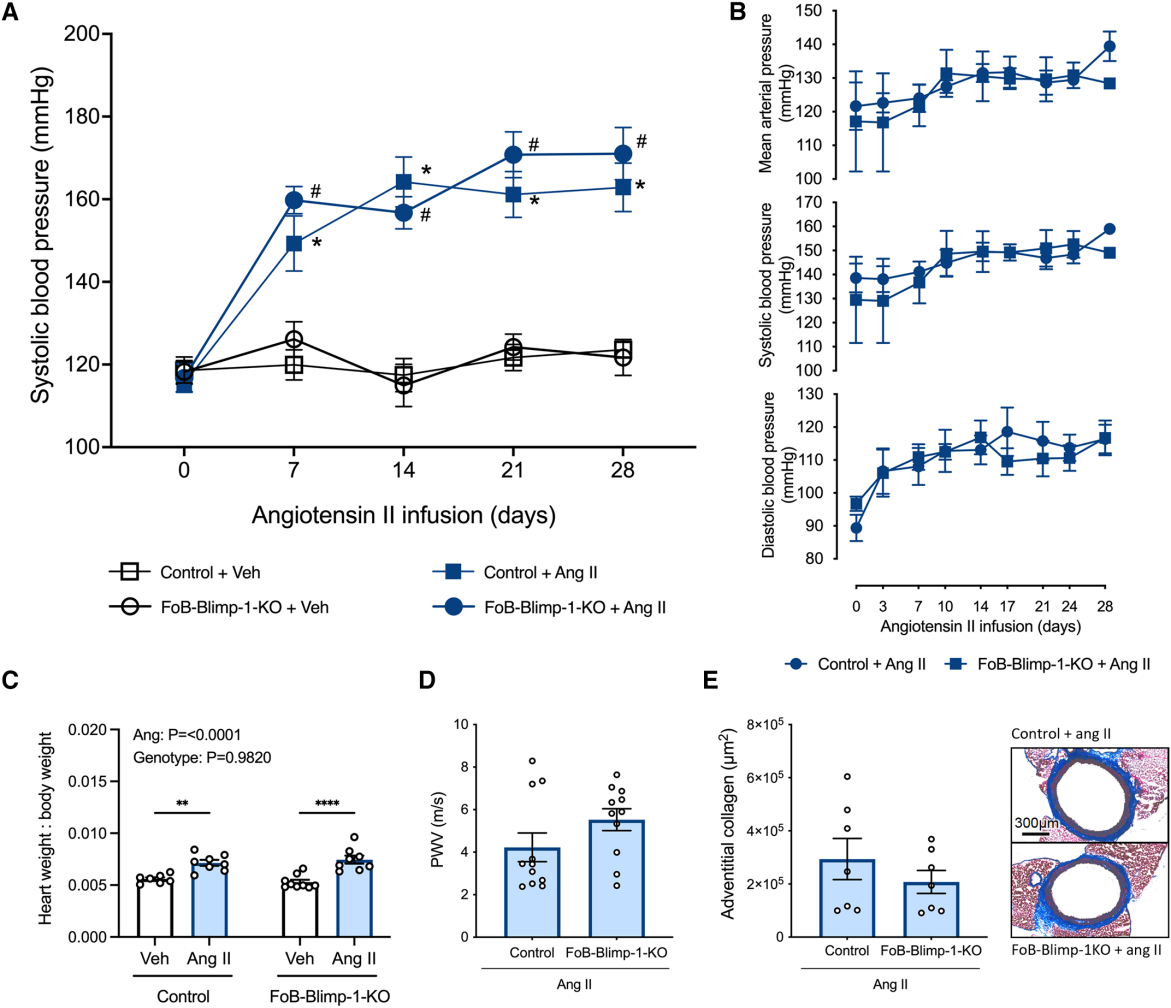
FoB-Blimp-1-KO mice were not protected from increases in blood pressure, cardiac hypertrophy, fibrosis or aortic stiffening. Blood pressures as measured by tail-cuff plethysmography (**A**) and radiotelemetry (**B**), heart weight to body weight ratio (**C**), pulse-wave velocity (PWV) (**D**) and adventitial collagen deposition (**E**) of vehicle (0.5% NaCl, 0.1% acetic acid) and angiotensin II (0.7 mg/kg/day) infused mice are shown. Data are represented as the mean ± standard error of the mean. For systolic blood pressure measurements (**A**), *indicates a *P* value <0.05 for Control + Veh group vs. the Control + Ang II group; ^#^indicates a *P* value <0.05 for FoB-Blimp-1-KO + Veh group vs. the FoB-Blimp-1-KO + Ang II group; For the heart weight to body weight ratio (**C**) data **P values <0.01 and ****P value <0.0001. Treatment effects are indicated at the top of the relevant bar graphs.

Tail-cuff plethysmography revealed that in vehicle-infused mice, systolic BP remained constant throughout the 28-day study period (Figure 4A). By contrast, angiotensin II infusion increased systolic BP within 7 days, reaching a plateau between days 14–21, and remaining at this heightened level for the remainder of the 28-day study period (Figure 4A). Blimp-1 truncation did not impact baseline systolic BP in vehicle-infused mice, nor did it influence the ability of angiotensin II to increase systolic BP at any of the timepoints post-surgery (Figure 4A). Similar findings were obtained by radiotelemetry whereby angiotensin II-induced increases in systolic, diastolic and mean arterial BP did not differ between control and FoB-Blimp-1-KO mice infused with angiotensin II (Figure 4B). Thus, the loss of Blimp-1 function in follicular B cells did not blunt angiotensin II-induced hypertension. This is in line with heart weight to body weight ratio (HW:BW) data which revealed FoB-Blimp-1-KO mice to exhibit similar development of cardiac hypertrophy as control mice infused with angiotensin II (Figure 4C). Furthermore, the truncation did not affect aortic stiffening as measured by pulse-wave velocity (Figure 4D) or fibrosis as measured by adventitial collagen staining (Figure 4E).

## 4 Discussion

In this study we sought to investigate whether the pro-hypertensive actions of B cells can be attributed to the commitment of follicular B cells to an ASC fate. For this, we used a mouse model with a selective Blimp-1 inactivating truncation driven by the CD23 expression of Cre recombinase (FoB-Blimp-1-KO mice). We showed that while the total number of B cells committed to an ASC fate were not altered in FoB-Blimp-1-KO mice, the truncation did reduce basal serum IgG1 and IgG2a levels, and prevented the increase in serum IgG3 following angiotensin II infusion, findings consistent with an impairment in the differentiation of follicular B cells into ASCs. Nevertheless, FoB-Blimp-1-KO and control mice infused with angiotensin II displayed similar increases in BP and cardiac hypertrophy. Moreover, after infusion with angiotensin II FoB-Blimp-1-KO and control mice exhibited similar aortic collagen deposition and vascular compliance. As FoB-Blimp-1-KO mice were still ASC sufficient, it is possible that other B cell subtypes may have compensated for the developmental block in follicular B cell-derived ASCs. Unfortunately, as ASCs downregulate the expression of virtually all B cell surface markers (46) it is difficult to establish whether other subtypes contributed to the remaining ASC pool. Thus, other B cell subtypes and/or antibody-independent functions (25–29, 34–38) of B cells may be responsible for the pro-hypertensive actions of these cells reported in previous studies (13–15, 21–23).

Studies using the FoB-Blimp-1-KO model have reported reductions in ASCs and total plasma IgG (26, 47). In the present study, baseline reductions in serum IgG1 and IgG2a were consistent with those findings. However, we did not observe a reduction in total ASCs in FoB-Blimp-1-KO mice. High expression of the cell surface marker CD138 is widely used to identify ASCs by flow cytometry (44), especially as CD138 expression is one of the first surface markers to be expressed by mature, activated B cells committed to an ASC fate (48). However, CD138 expression is not exclusively expressed on ASCs and may also be present on developing pre-B cells (49). A study using GFP reporter mice (44) showed ASCs are better identified by the co-expression of CD138 and Sca-1, where nearly 100% of cells within this gate expressed Blimp-1. Thus, the discrepancy between (26, 47) and the present study may be explained by differences in gating strategies where the former identified ASCs as CD19^−^CD138^hi^ cells whereas we used the more stringent approach of including Sca-1. Nevertheless, given that follicular B cells are classically associated with the production of high-affinity class-switched antibodies (33), the lower baseline levels of serum IgG1 and IgG2a provide at least indirect evidence that their differentiation into ASCs was impaired in our model. This is supported by another study showing that while Blimp-1 was not required for initial B cell commitment to an ASC fate, its expression was necessary for their terminal differentiation and high-capacity antibody secretion (50). The findings from that study combined with our more selective approach of identifying ASCs may, at least in part, explain the lower levels of serum IgG isotypes despite apparently similar ASC numbers compared to controls.

Since the ASC population is composed of both plasmablasts and plasma cells, we sought to investigate whether selective Blimp-1 knockout or angiotensin II infusion differentially affected these subpopulations. Consistent with their lack of an effect on total ASC numbers, FoB-Blimp-1-KO mice did not exhibit fewer plasmablasts or plasma cells in the spleen or bone marrow than control mice. Similarly, infusion with angiotensin II did not affect the total number of ASCs, plasmablasts or plasma cells in the spleen or bone marrow of FoB-Blimp-1-KO or control mice. These findings are consistent with previous work (23) and recent work from our group (42) in which there were no increases in ASCs following angiotensin II infusion.

Innate-like B-1 (B-1a and B-1b) and conventional B-2 (marginal zone and follicular) B cells are the two major B cell lineages which emerge during embryonic and foetal development, respectively (51). B-1 cells are regarded as the main source of low-affinity polyreactive serum IgM and IgA (52, 53), sometimes referred to as “natural” antibodies (53). In contrast to B-2 cells, B-1 cells are devoid of CD23 expression (39). Hence, it is not surprising that serum IgM and IgA were not altered by FoB-Blimp-1-deletion. We have previously reported that angiotensin II infusion increases serum IgG3 in wild-type mice (21). Here, angiotensin II infusion similarly increased serum IgG3 in control mice, but not in FoB-Blimp-1-KO mice, suggesting a role for follicular B cell-derived ASCs in IgG3 production during hypertension. IgG3 is known to be robustly elevated following T cell-independent antigen stimulation (54), primarily by B-1 and marginal zone B cells (55, 56). However, mature recirculating follicular B cells, which migrate and occupy perisinusoidal niches in the bone marrow, can respond to T cell-independent antigens in response to blood-borne infections (57). Thus, it is possible that follicular B cell activation following angiotensin II infusion is, at least partially, driven by T cell-independent antigens. Alternatively, the transient expression of CD23 during marginal zone B cell development (58), and therefore potentially Cre recombinase, may have impaired the secretion of marginal zone-derived antibodies and/or their differentiation into short-lived plasmablasts (59–62). Recent studies have shown that the downregulation of the B cell transcriptional program and upregulation of the plasma cell transcriptional program relies on a series of coordinated, epigenetic chromatin remodelling events (63–65). Given that chromatin accessibility is known to impact the ability of Cre recombinase to access *loxP* sites (66), Cre recombinase may have had limited access to the floxed Blimp-1 locus during marginal zone B cell development. Thus, future studies should investigate whether a CD23-driven truncation of Blimp-1 impairs marginal zone B cell function and/or differentiation into ASCs. Nevertheless, since a reduction in IgG3 did not reduce SBP in hypertensive mice, it is unlikely that IgG3 secretion contributes to BP dysregulation associated with the condition.

The present study highlights that the differentiation of follicular B cells into ASCs and/or the secretion of IgG1, IgG2a and IgG3 may not be the key effector mechanisms mediating the pro-hypertensive actions of B cells. Given that FoB-Blimp-1-KO mice were ASC sufficient, it is possible that other B cell subtypes differentiated into ASCs and compensated for the genetic block in follicular B cell-derived ASCs. Notwithstanding potential sex-specific differences and other B cell functions such as antigen presentation and cytokine secretion, future studies should consider the contribution of other B cell subsets to hypertension as these have distinct roles and may respond differently to hypertensive stimuli.

## Data Availability

The original contributions presented in the study are included in the article/**Supplementary Material**, further inquiries can be directed to the corresponding author.
