# Supplementary material for: Depletion of follicular B cell-derived antibody secreting cells does not attenuate angiotensin II-induced hypertension or vascular compliance
Source: Front Cardiovasc Med. 2024 May 31;11:1419958. doi: 10.3389/fcvm.2024.1419958 (PMC11176447; doi:10.3389/fcvm.2024.1419958)
Supplement: Supplementary file 1 [file Datasheet1.docx]

Supplementary Material

# Supplementary Figures and Tables

## Supplementary Figures


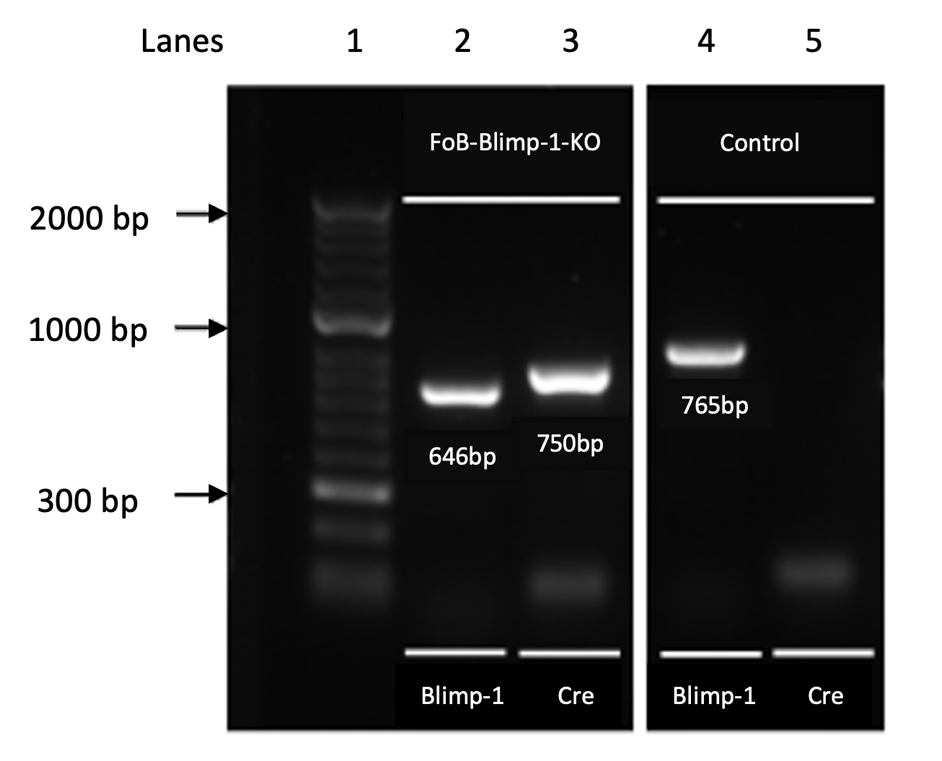


Supplementary Figure 1. Confirmation of Blimp-1 truncation in B cells. Representative gel electrophoresis blot of PCR products from PCR genotyping of splenic B cell DNA isolated from untreated control (*Prdm1*^fl/fl^: lanes 2-3) and FoB-Blimp-1-KO (*Fcer2a-Cre*^+/-^*Prdm1*^fl/fl^; lanes 4-5) mice. Positive identification of: floxed Blimp-1 allele (765 bp) in lane 2; exon-6-deleted Blimp-1 allele (646 bp) in lane 4; and Cre recombinase allele (750 bp) in lane 5. Lane 1 contains the DNA ladder used for band identification.

**
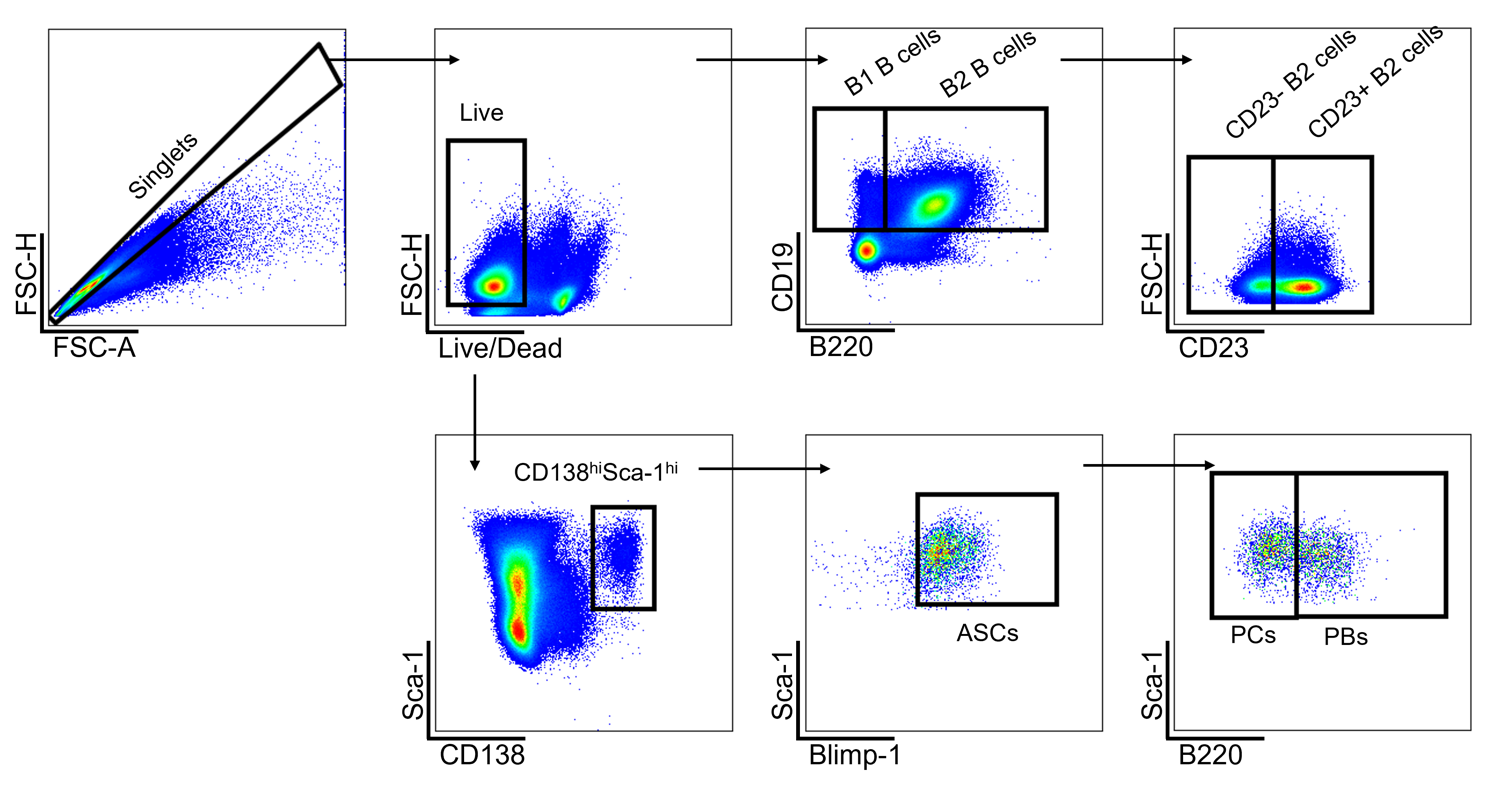
**

Supplementary Figure 2. Flow cytometry gating strategy used to enumerate B cells and antibody secreting cells (ASCs). Representative plots of the gating strategy used to quantify B-1 B cells (CD19^+^B220^-^), CD23^+^ and CD23^-^ B-2 B cells (CD19^+^B220^+^), ASCs (CD138^hi^Sca-1^+^Blimp-1^+^), plasmablasts (PBs; CD138^hi^Sca-1^+^Blimp-1^+^B220^+^) and plasma cells (PCs; CD138^hi^Sca-1^+^Blimp-1^+^B220^-^) from 1% formalin fixed spleen and bone marrow samples.


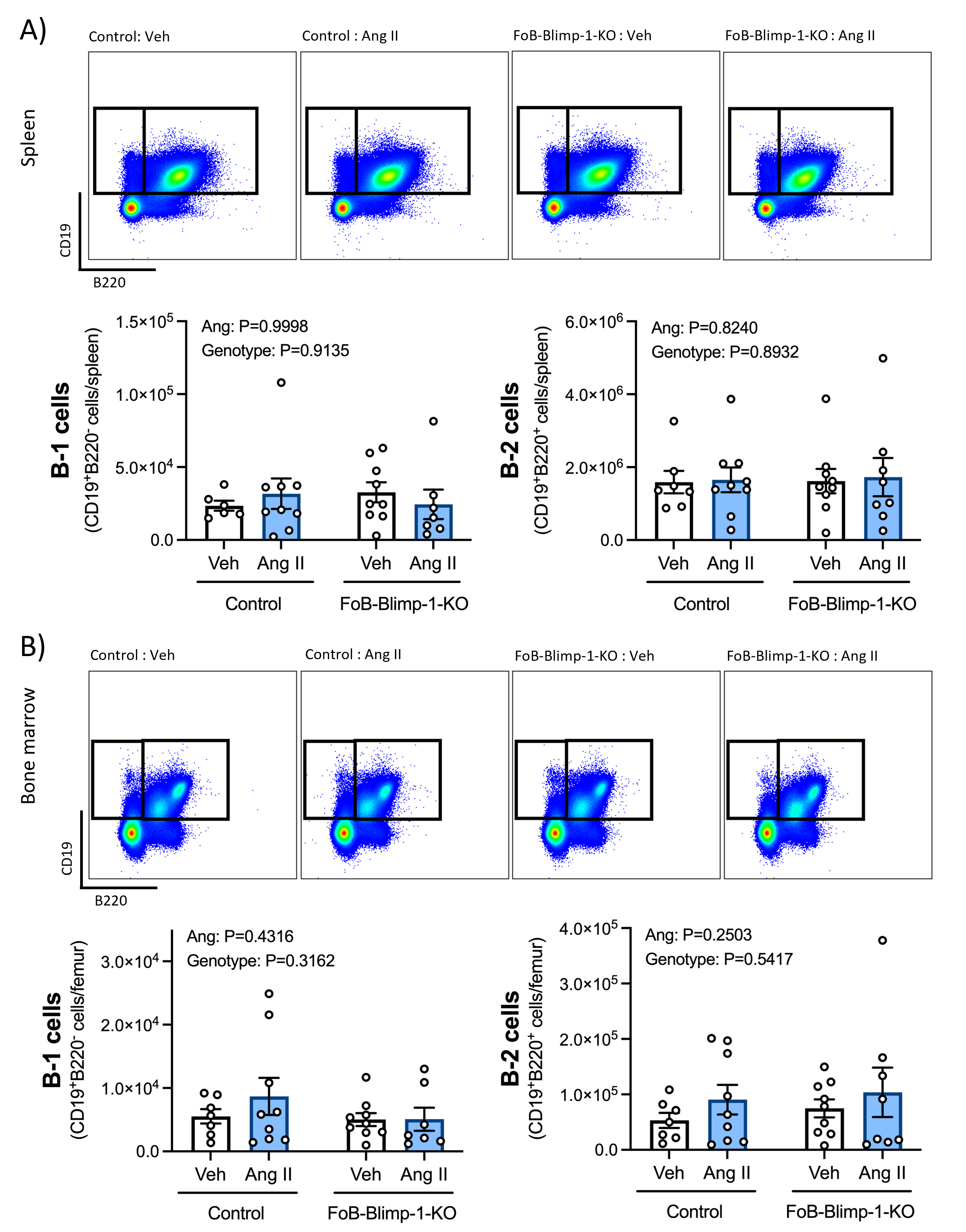


Supplementary Figure 3. Follicular B cell Blimp-1 truncation and angiotensin II infusion do not affect B-1 or B-2 B cell numbers in the spleen or bone marrow. Representative flow cytometry plots and bar graphs depicting the effect of follicular B cell Blimp-1 truncation on B-1 and B-2 cells in the spleen (panel A) and bone marrow (panel B). B-1 B cells were defined CD19^+^B220^-^ and B-2 B cells as CD19^+^B220^+^ cells gated from all live splenocytes or all live bone marrow derived cells. Data are represented as the mean ± standard error of the mean of the total cell counts per spleen or per femur. Treatment effects are indicated at the top of the graphs.


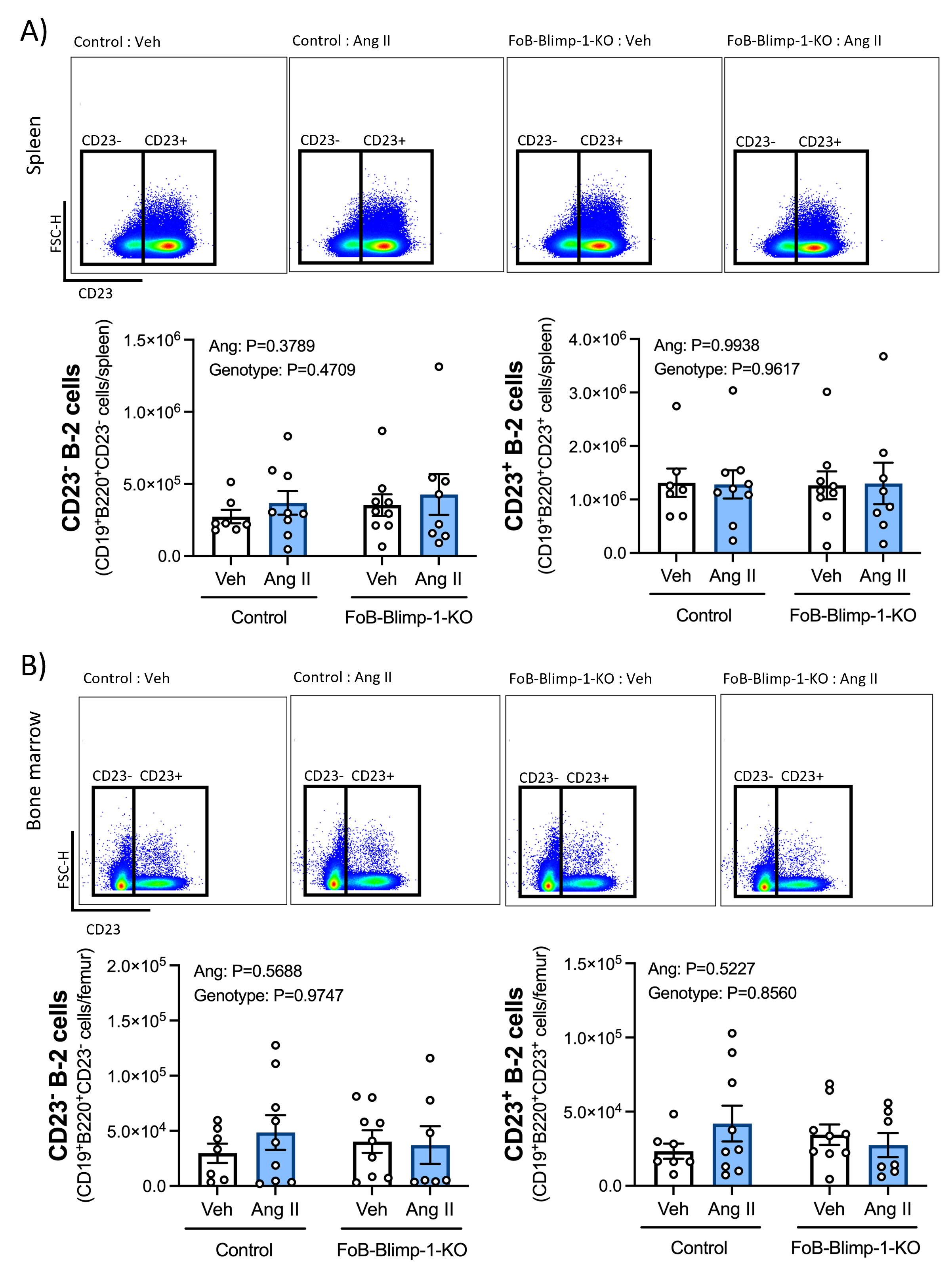


Supplementary Figure 4. CD23+ and CD23- B-2 B cell numbers are not affected by follicular B cell Blimp-1 truncation or angiotensin II infusion. Representative flow cytometry plots and bar graphs depicting the effect of follicular B cell Blimp-1 truncation on CD23^+^ and CD23^-^ B-2 B cells in the spleen (panel A) and bone marrow (panel B). B-2 B cells were defined as CD19^+^B220^+^ cells gated from all live splenocytes or all live bone marrow derived cells, prior to their separation into CD23^+^ and CD23^-^ populations. Data are represented as the mean ± standard error of the mean of the total cell counts per spleen or per femur. Treatment effects are indicated at the top of the graphs.
